# Serotonin Syndrome in the Setting of Lamotrigine, Aripiprazole, and Cocaine Use

**DOI:** 10.1155/2015/769531

**Published:** 2015-08-02

**Authors:** Anupam Kotwal, Sarah L. Cutrona

**Affiliations:** ^1^Internal Medicine, University of Massachusetts Medical School, 55 Lake Avenue North, Room H6-531, Worcester, MA 01655, USA; ^2^Division of General Medicine and Primary Care, Meyers Primary Care Institute, University of Massachusetts Medical School, 365 Plantation Street, Biotech 1, Suite 100, Worcester, MA 01655, USA

## Abstract

Serotonin syndrome is a potentially life-threatening condition associated with increased serotonergic activity in the central nervous system. It is classically associated with the simultaneous administration of two serotonergic agents, but it can occur after initiation of a single serotonergic drug or increasing the dose of a serotonergic drug in individuals who are particularly sensitive to serotonin. We describe a case of serotonin syndrome that occurred after ingestion of higher than prescribed doses of lamotrigine and aripiprazole, in addition to cocaine abuse. The diagnosis was established based on Hunter toxicity criteria and severity was classified as mild. The features of this syndrome resolved shortly after discontinuation of the offending agents. Serotonin syndrome is characterized by mental status changes, autonomic hyperactivity, and neuromuscular abnormalities along a spectrum ranging from mild to severe. Serotonin syndrome in our patient was most likely caused by the pharmacokinetic and pharmacodynamic interactions between lamotrigine, aripiprazole, and cocaine leading to increased CNS serotonergic activity.

## 1. Introduction

Serotonin syndrome is a potentially life-threatening condition associated with increased serotonergic activity in the central nervous system (CNS). It is classically associated with the simultaneous administration of two serotonergic agents, but it can occur after initiation of a single serotonergic drug or increasing the dose of a serotonergic drug in individuals who are particularly sensitive to serotonin [1–4]. Lamotrigine, aripiprazole, and cocaine have traditionally not been associated with this syndrome and none of them have strong serotonergic activity by themselves. An extensive literature search on PubMed did not yield any case description of serotonin syndrome induced by lamotrigine, aripiprazole, and cocaine alone or in combination. Also, UpToDate, Micromedex, and Epocrates do not list this syndrome as an adverse effect for any of these agents. We hypothesize that pharmacokinetic and pharmacodynamic interactions between these agents were responsible for inducing serotonin syndrome in our patient.

## 2. Case Presentation

A 24-year-old Caucasian female with a psychiatric history of bipolar disorder, posttraumatic stress disorder (PTSD), and cocaine abuse was admitted for nausea, dizziness, and jitteriness that started after intentional ingestion of 4 gm of lamotrigine and 80 mg of aripiprazole, in addition to cocaine abuse. On admission, she was alert, oriented, and afebrile but was noted to be diaphoretic, tachycardic, and in mild distress due to nausea and vertigo. Her pupils were equal, round, and reactive to light but horizontal nystagmus was present in both eyes. Neurologic examination was remarkable for hyperreflexia in bilateral lower extremities with inducible patellar and ankle clonus but normal sensation and strength. Laboratory studies were remarkable only for mildly elevated liver enzymes that normalized within 24 hours. Urine was positive for codeine, cocaine, and lamotrigine. On the date of admission, blood level of lamotrigine was 7.5 mcg/mL (reference 3–14) and that of aripiprazole was 760 ng/mL (reference 0–870), both being within normal limits.

Based on the Hunter criteria, she was diagnosed with serotonin syndrome with severity classified as mild. Other differential diagnoses were ruled out based on clinical grounds and medication history. She was managed by discontinuing lamotrigine and aripiprazole and with oral lorazepam for symptom control for a period of 24 hours. During the hospital stay, her tachycardia and nystagmus resolved over 24 hours. The clonus and hyperreflexia improved and later resolved over 48 hours. Her symptoms of nausea, dizziness, jitteriness and diaphoresis also resolved within 24 hours. Her skin remained completely normal during and after hospital stay; this was monitored because of concern for Stevens-Johnson syndrome with lamotrigine.

After the resolution of features consistent with serotonin syndrome, the patient was started on divalproex and risperidone for bipolar disorder and PTSD. On follow-up, her psychiatric conditions have been well managed. She has continued to abuse cocaine but has not developed similar symptoms or signs again.

## 3. Discussion

Serotonin syndrome is characterized by mental status changes, autonomic hyperactivity, and neuromuscular abnormalities along a spectrum ranging from mild to severe [1, 5–7]. Mental status changes can include anxiety, agitated delirium, restlessness, and disorientation. Autonomic manifestations can include diaphoresis, tachycardia, hyperthermia, hypertension, nausea, vomiting, and diarrhea [1, 5, 6]. Neuromuscular hyperactivity usually manifests as hyperreflexia and clonus most pronounced in the lower extremities but may present as muscle rigidity and bilateral Babinski sign [1]. Laboratory abnormalities in severe cases can include metabolic acidosis, rhabdomyolysis, elevated levels of serum aminotransferase and creatinine, renal failure, and disseminated intravascular coagulopathy [1]. The onset of symptoms is usually rapid, and resolution does not occur unless there is discontinuation of the offending agent(s).

The Hunter criteria are most widely used for the diagnosis of this syndrome (84% sensitivity and 97% specificity) [1, 8]. The diagnosis is established if the patient has taken a serotonergic agent and meets any of the following criteria: spontaneous clonus, inducible clonus plus agitation or diaphoresis, ocular clonus plus agitation or diaphoresis, tremor plus hyperreflexia, and hypertonia plus temperature above 38°C plus ocular clonus or inducible clonus [8]. Differential diagnoses including anticholinergic poisoning, malignant hyperthermia, neuroleptic malignant syndrome, and sympathomimetic toxicity can be distinguished from serotonin syndrome on the basis of clinical features and medication history. In our patient, neuroleptic malignant syndrome was unlikely given the acute nature of symptoms and the absence of rigidity, hyperthermia, or altered mental status. Anticholinergic toxicity was unlikely given the presence of hyperreflexia and the absence of other anticholinergic features. Malignant hyperthermia and other causes of agitated delirium like CNS infections or sympathomimetic toxicity were also very unlikely. The rapid resolution of signs and symptoms after discontinuation of the offending agents also pointed towards serotonin syndrome as the likely diagnosis.

Stimulation of the postsynaptic 5-HT1A and 5-HT2A receptors has been implicated, but this syndrome may result from any combination of drugs that has the net effect of increasing serotonergic neurotransmission [1, 9]. The selective serotonin reuptake inhibitors (SSRIs) are perhaps the most commonly implicated group of medications associated with serotonin syndrome. Our patient developed features of serotonin syndrome after ingestion of higher than prescribed doses of lamotrigine and aripiprazole, in addition to cocaine abuse. However, none of these agents have strong serotonergic activity by themselves, and the blood levels of lamotrigine and aripiprazole in our patient were within normal limits. These agents do have some effect on serotonin neurotransmission. Lamotrigine has a weak inhibitory effect on 5-HT_3_ receptor, aripiprazole is partial agonist at 5-HT_1A_ receptor and an antagonist at serotonin reuptake transporter, and cocaine increases the release and inhibits the reuptake of serotonin at the synaptic cleft. Serotonin syndrome in our patient was most likely caused by the pharmacokinetic and pharmacodynamic interactions between lamotrigine, aripiprazole, and cocaine leading to increased CNS serotonergic activity.

## 4. Conclusions

The pharmacokinetic and pharmacodynamic interactions between lamotrigine, aripiprazole, and cocaine can lead to increased CNS serotonergic activity. Hence, serotonin syndrome should be considered in the differential of a patient who takes these medications and demonstrates even subtle features of neuromuscular hyperactivity. The management of serotonin syndrome includes discontinuation of the offending agents, supportive care, and benzodiazepines for symptom control, with serotonin antagonists being a last resort.

## Abbreviations

5-HT: 5-Hydroxytryptamine
CNS: Central nervous system
PTSD: Posttraumatic stress disorder
SSRI: Selective serotonin reuptake inhibitor.
